# Quantitative high-throughput population dynamics in continuous-culture by automated microscopy

**DOI:** 10.1038/srep33173

**Published:** 2016-09-12

**Authors:** Jason Merritt, Seppe Kuehn

**Affiliations:** 1 Center for the Physics of Living Cells, University of Illinois at Urbana-Champaign, Urbana, IL 61801, USA; 2 Department of Physics, University of Illinois at Urbana-Champign, Urbana, IL 61801, USA; 3 Center for Biophysics and Quantitative Biology, University of Illinois at Urbana–Champaign, Urbana, IL 61801, USA

## Abstract

We present a high-throughput method to measure abundance dynamics in microbial communities sustained in continuous-culture. Our method uses custom epi-fluorescence microscopes to automatically image single cells drawn from a continuously-cultured population while precisely controlling culture conditions. For clonal populations of *Escherichia coli* our instrument reveals history-dependent resilience and growth rate dependent aggregation.

Continuous-culture methods have been widely used to maintain microbial populations and communities in precisely controlled conditions. Continuous-culture devices are used to study metabolic processes^1^, evolutionary dynamics including antibiotic resistance^2^, phage-bacteria coevolution^3^, adaptive radiation^4^, predator-prey interactions^5^ and mutualisms^6^.

All of these applications of continuous-culture devices require the measurement of microbial abundances. However, quantitative automated techniques for measuring abundances remain limited. Most abundance measurements are made by sampling and plating^3^ or hemacytometry^5^; but sampling methods are labor-intensive, subject to uncontrolled bias and have poor temporal resolution. More sophisticated methods to measure abundances include optical density measurements^2^, flow cytometry^7^, digital holography^8^,^9^ and light scattering measurements^10^. Automated optical density measurements are high-throughput and cost-effective but are indirect measures of abundance that can vary with cell size or shape, and have a small dynamic range. Flow cytometry solves these problems, but is challenging to automate and requires costly lasers and sensitive light detection hardware. Another technique for measuring abundances in microbial populations based on fluctuations in the intensity of scattered light^10^ was recently demonstrated; however, this approach does not image single cells directly, requires precise alignment of optical components and is sensitive to changes in cell morphology. Digital holography has also been used to make long term measurements of abundance dynamics in complex microbial communities^8^,^9^. Holography can distinguish species morphologically but cannot separate species or strains using fluorescence and is computationally demanding.

To address these problems, we have developed broadly applicable, low-cost, automated continuous-culture devices coupled to custom-built epi-fluorescence microscopes. These devices measure abundance dynamics in bacterial communities through automated single-cell imaging. Our instrument provides complementary capabilities to light scattering^10^, holography^8^,^9^ and flow cytometry. The advantages of our instrument are that single-cell fluorescence imaging does not require morphologically distinct species, is immune to artifacts from light scattering by detritus or aggregates and requires little precise optical alignment, allowing stable acquisition for many weeks. Further, we retain precise control over culture conditions.

Using this device we show that clonal populations of *Escherichia coli* exhibit historical dependence in the resilience of the population to perturbations and complex, growth rate dependent aggregation. The throughput of our measurement permits us to statistically characterize these processes in a simple microbial community.

## Results

**Figure 1:**
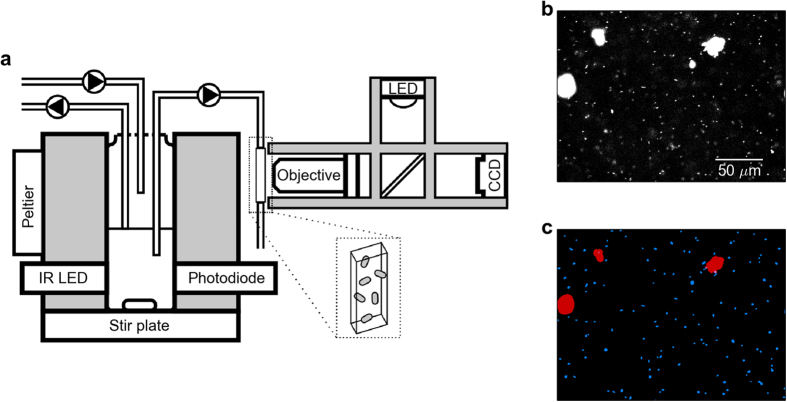
Microscope-coupled continuous-culture device and image segmentation. **(a)** Continuous-culture device and associated epi-fluorescence microscope. System temperature is set by a Peltier element and feedback to a thermometer (not shown), and optical density is measured by an infrared LED and photodiode in an absorbance geometry. The culture vial is stirred with an inductive stir plate, fed by a nutrient inflow line, and the culture volume is set by the outflow line. A separate microscope imaging line pumps culture samples into a thin rectangular glass capillary (inset) centered on the microscope’s focal plane for imaging. The fluorescence microscope uses an epi-fluorescence geometry with a high-powered LED, dichroic mirror, objective, and CCD camera. (**b**) Example image from device during operation, showing single cells and large cell aggregates or ‘clumps’ from *E. coli* constitutively expressing red fluorescent protein (dTomato). Contrast and brightness have been increased to show out-of-focus as well as in-focus objects. (**c**) Segmented image showing regions determined to be in-focus cells (blue) and clumps (red) by our custom image segmentation software (Methods).

We constructed six replicate continuous-culture devices (Fig. 1a). Each system houses a 20 mL culture in a glass vial in a custom machined aluminum block. The continuous dilution of the culture is accomplished by two computer-controlled peristaltic pumps which add fresh media and remove organisms and spent media. The volume of the culture is fixed by the height of the outflow line relative to the bottom of the vial. The culture vial is in thermal contact with the aluminum block, which is temperature controlled via computer-controlled PID feedback to a thermoelectric heating-cooling element (Peltier). The optical density of the culture is monitored using an infrared light-emitting diode (LED) and photodetector mounted in the aluminum block. A magnetic stir bar and commercial inductive stir plate mix the culture.

During continuous-culture, a third peristaltic pump draws samples once per minute from the culture vial and passes it through a micron-scale glass capillary (Fig. 1a). A custom single-color epi-fluorescence microscope images cells as they pass through the capillary before being returned to the culture vial. Within the imaging volume, the sample is illuminated by filtered light from a high-power LED. The resulting fluorescence is captured by a 20x objective and imaged by a charge-coupled device (CCD) image sensor. We found glass capillaries connected directly to silicone tubing with medical epoxy to be far more robust than Polydimethylsiloxane microfluidic devices. Metal extrusions supporting tubes that pass samples from the culture vial to the microscope were necessary to damp vibrations. Bubbles infiltrate our imaging line, but only transiently remain in the capillary and the system remains resilient to their presence over periods of weeks. The total volume of the tubing between the culture vessel and the capillary is small (~0.5 mL) to minimize the population held outside the vial and facilitate rapid sampling of cells from the culture.

Image acquisition occurs after a short pulse from the peristaltic pump passes a fresh sample through the capillary. When fluid flow slows (approximately 20 seconds) we acquire 5 images in one second with an exposure time of 25–150 ms depending on the experiment. The images are segmented in real-time by custom software (Fig. 1b,c). To segment the images, a difference of Gaussians filter (http://scikit-image.org/) detects bright objects, followed by local thresholding in a small region around each object and a region growing technique modified from Lindeberg’s blob detection algorithm^11^. The segmentation routines cannot distinguish between in-focus and out-of-focus objects, so segmented objects are further classified using a support vector machine (SVM) trained on a manually-classified set of in-focus and out-of-focus cells. Separately, objects much too large to be individual cells – including filamentous cells and large cell aggregates – are detected by global moments-preserving thresholding^12^ on the entire image (https://github.com/fiji/Auto_Threshold). Finally, low-quality images are rejected automatically by thresholding on statistical properties of the spatial distributions of objects in the imaging volume (Methods).

We tested the long-term stability of our chemostat and imaging apparatus by running three replicate systems at a constant dilution rate (0.08 h^−1^) for 550 hours. We found the abundance dynamics across replicates to be reproducible (Supplementary Fig. S4a), demonstrating the long-term stability of our chemostats and microscopy.

**Figure 2:**
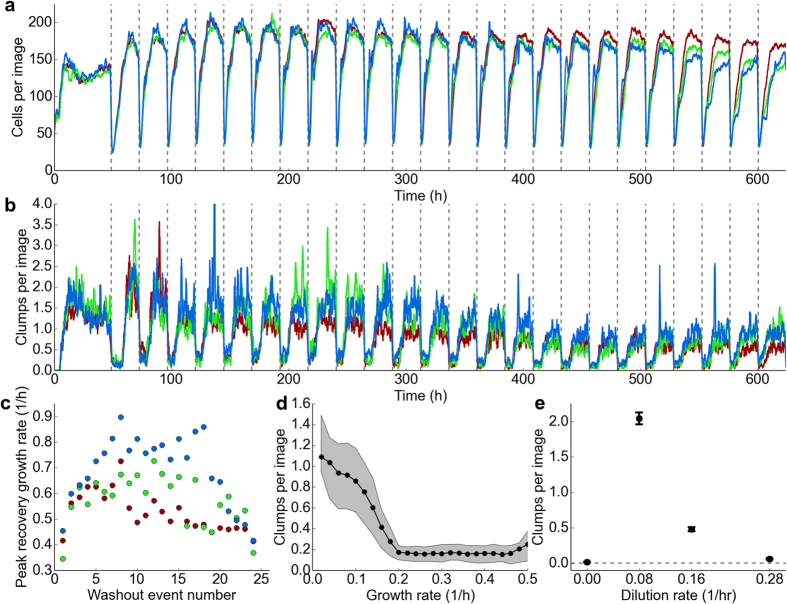
Abundance and aggregation dynamics in fluctuating nutrient conditions. (**a**) Single-cell abundances, not including aggregates, from image segmentation in three replicate continuous-culture devices over a 26-day experiment. Data shown is smoothed by a rolling average with a one-hour window. Each dashed vertical line represents a washout event where ~90% of the culture is pumped out and replaced with fresh media. **(b)** Clump abundances for three replicate systems during the same experiment shown in (**a**). Data shown is smoothed by a one-hour rolling average. **(c)** Peak population recovery rates (Supplementary Fig. S1) increase quickly after the first washout event, but eventually decline. Peak growth rates are calculated during single-cell population recovery (Methods) following the washout events shown in (**a**). Error bars from fit are smaller than markers. **(d)** Following a washout event, clump abundances remain low until the specific growth rate of the single-cell population slows to approximately 0.2 h^−1^. Graph shows data from recovery after all washout events in one system, before the next washout event begins, sorted into equal-width growth rate bins. Shaded region show central two-thirds of clump abundance data in each bin. (**e**) Separate experiments were carried out to determine clump abundances after a population reaches steady state at a constant dilution rate, rather than during recovery after a washout event. Data represents a time average of clump abundances between 40 and 48 hours after a population’s introduction to constant dilution conditions, and error bars show the standard error of the mean for data in this period.

We used this system to study long-term abundance dynamics in communities of *Escherichia coli* in fluctuating nutrient conditions, where the population alternates between periods of slow growth (chemostat dilution rate, D = 0.08 h^−1^, doubling time, t_d_ = 8.6 hours) and rapid (1 hour) dilution of 90% of the population and replenishment of nutrients (“washout” event). We performed this experiment in minimal media with low glucose concentrations (0.04% w/v, 2.2 mM). During the slow growth phase we expect the glucose concentration in the culture medium to be in the micro-molar range^13^. The washout events increase glucose concentrations in the culture to approximately 2 mM driving rapid growth until abundances return to their steady-state value (Fig. 2a). Immediately after the washout event the dilution rate of the chemostat returns to 0.08 h^−1^. After an initial 48-hour acclimatization period (D = 0.08 h^−1^) we applied washout events every 24 hours for a period of 26 days in three replicate continuous-culture devices.

We observe that the resilience of the population, defined as the maximum rate of recovery from washout events (Supplementary Fig. S1), depends on the history of past perturbations (Fig. 2c). In particular, the resilience of the population increases approximately 50% over the first 3–4 washout events. Since the nutrient concentrations must be nearly identical after each washout event, this behavior cannot be ascribed to variable nutrient concentrations in the culture during the recovery. Further, this increasing resilience is apparent after just 16 generations (120 hours) which is very likely too fast for a mutation to fix. For example, a mutant with a selection coefficient 
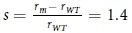
 (where *r*_*m*_ and *r*_*WT*_ are the mutant and wild-type growth rates respectively) would reach a relative abundance of approximately 0.5 on this timescale. A selection coefficient of 1.4 is 10 to 100 times larger than typically observed for bacterial chemostats^14^. Previous studies of *E. coli* under glucose starvation conditions have shown that *rpoS* mutants can reach high abundance on the timescale of 10 generations, but such mutants do not arise in the MG1655 genetic background used here^4^. However, at long times (>200 h), genetic diversity likely becomes significant^4^, and this diversity may account for the decline in resilience and increasing variation across replicate communities we observe (Fig. 2c).

Therefore, we conclude the time-dependent resilience we observe over the first 6 days of the experiment is driven by non-genetic changes in the population. Such changes may include metabolic adaptation to the fluctuating environment that is uniform across the population such as history dependent lag-phase^15^. Another possibility is the presence of a slow-growing subpopulation, such as persisters^16^,^17^. If a large fraction of the population is slow-growing prior to the first washout event, and subsequently declines with repeated washout events this could account for increasing resilience^17^. Discriminating between these possibilities will require growth measurements at the single-cell level.

Our instrument also reveals growth rate dependent aggregation (Fig. 1b). To our knowledge the presence of aggregates in slow-growing populations of *E. coli* has not been previously reported, likely due to the difficulty in discerning their presence using standard techniques such as plating and optical density measurements. These aggregates, or ‘clumps,’ typically contain tens of cells and can make up approximately half of the biomass present in a community (Methods). We observe surprising aggregation dynamics over the course of a washout event (Fig. 2b,d). Immediately after a washout event aggregates remain at low abundance as the planktonic cell abundances increase rapidly (growth rates >0.3 h^−1^). When the growth rate of single cells declines to approximately 0.2 h^−1^, we observe a rapid rise in the presence of aggregates (Fig. 2d).

We performed additional short-term experiments to investigate the growth rate dependence of aggregation. In a chemostat the growth rate must equal the dilution rate at steady state^14^. We operated the chemostat at different dilution rates (growth rates): D = 0 (batch culture), 0.08 h^−1^ (t_d_ = 8.6 h), 0.16 h^−1^ (t_d_ = 4.3 h) and 0.28 h^−1^ (t_d_ = 2.5 h) without applying washout events and quantified the abundance of clumps after the abundance of single cells reached steady state (40–48 hours, Supplementary Fig. S2). These measurements revealed a surprising non-monotonic dependence of aggregation on growth rate (Fig. 2e). We observe no aggregation as a batch culture enters stationary phase (zero growth), a peak in the number of aggregates at D = 0.08 h^−1^ and a steady decline in the number of aggregates in the chemostat up to D = 0.28 h^−1^ (for detailed description of control experiments see Supplementary Information).

Cellular aggregation in the form of attached biofilms or ‘wall growth’ has been widely observed in chemostats^14^. It is possible that the aggregates we observe result from the release of attached biofilms; however, the clumps in our experiments manifest within the first generation, are suppressed at higher growth rates (where adhesion is more likely^14^), and decline in abundance over time, making wall growth an unlikely source. Also, for the experiment in Fig. 2a we observe minimal adherence to the vial walls, tubing and capillary after 26 days of continuous culture. We performed additional experiments to show that aggregates formed at slow growth rates with or without selective antibiotics (our *dTomato* expressing strain is chloramphenicol resistant) and mutants with reduced adhesion (Δ*fimA*, Δ*flu*^18^, Supplementary Fig. S3). We conclude that aggregation is triggered by the onset of very slow but non-zero growth rates (Fig. 2e).

Aggregates constitute surprising spatial structure present even in clonal populations under controlled environmental conditions. This structure has a complex, non-monotonic dependence on the growth rate of the population (Fig. 2e) and likely has important ecological implications since clusters of cells have elevated antibiotic resistance^19^ and are believed to concentrate nutrients locally through advective flow^20^.

## Discussion

We have demonstrated a low-cost, custom continuous-culture device with automated single-cell imaging to measure abundances that is robust for periods of weeks. Our instrument shows *E. coli* populations subject to fluctuating nutrient conditions exhibit time-dependent resilience and growth-rate dependent aggregation. Furthermore, the flexibility and simplicity of our system will permit it to be used for studying a range of questions, such as evolution in the presence of phage^3^ or the role of persisters in population dynamics^17^. Simple modifications to the current design will allow multicolor fluorescent imaging of population dynamics in an ecosystem of multiple species where quantitative measurements would permit the study of complex ecological interactions^9^.

## Methods

### Continuous-culture device control

Interfacing between a computer and control electronics was accomplished using a LabJack U3-LV DAQ device (LabJack). All systems except the camera were controlled by custom Python software using LabJackPython. Control of pumps and LEDs was accomplished through a low-cost 8-channel relay module (Kootek). Heating and cooling for thermal control of the culture was accomplished by driving a 12711-5L31-03CQ thermoelectric heating cooling element (Custom Thermoelectric) with a power supply and a VNH2SP30 motor driver (Pololu). Camera control was managed by directly interfacing with the camera using the FlyCapture software development kit (SDK, Point Grey).

### Liquid handling and stirring

Our continuous-culture device design is similar to that of Toprak *et al*.^2^, and we used 40 mL CG-4902-08 glass vials (ChemGlass) to house the 20 mL culture. All liquid connections to the cultures (inflow, outflow, microscope imaging lines) used combinations of 1 mm ID platinum-cured silicone tubing (VWR), 1.6 mm OD PEEK tubing (Fisher Scientific), and plastic connectors and luer fittings (Nordson Medical). Tubes were mounted into the culture vials using a custom 3D-printed autoclavable nylon ‘cap’ (Stratasys). We used 400 F/A single-channel peristaltic pumps (Watson-Marlow) with relay modules to control liquid flow, with matching Watson-Marlow ‘tube elements’ to fit the pumps: 1.02 mm ID 049.EBJA.102 silicone tube elements for basic liquid inflow and outflow, and 0.76 mm ID 049.EBJA.076 silicone tube elements for the microscope imaging line. At an acquisition frequency of once per minute the 0.5 mL microscope line is completely replenished at least every 10 minutes, a timescale short compared to the dilution rate of the continuous-culture device. For stirring, we used motor-free inductive Cimarec i Mono Direct stir plates (Fisher Scientific) calibrated using a custom Hall probe. All cultures were stirred at 800 rpm.

### System mounting and electronics

Custom machined parts (eMachineShop) designed to fit our culture vials were used to mount our systems to optical tables. For temperature measurement, we used EI1034 Temperature Probes with signal amplification from LJTick-InAmps (LabJack). Thermometers were independently calibrated using a high-accuracy digital thermometer. For optical density measurements, we used absorption geometry with TSAL6100 infrared (IR) emitters and BPV10NF IR photodiodes (Digi-Key) epoxied into nylon screws and mounted in our machine parts.

### Microscope design

Six identical custom single-color epi-fluorescence microscopes were constructed using Thorlabs optomechanics, lenses and stages in a 30 mm cage system mounted to an optical table, with an MA845 20x, 0.65 NA objective (Meiji Techno) mounted in an SM1Z linear stage (Thorlabs) for focusing. The field of view was approximately 240 μm × 160 μm. Excitation illumination was provided by a high-power lime M565D2 LED (Thorlabs) controlled by a relay module and filtered using 49004 dTomato-appropriate filter-dichroic sets (Chroma). Illumination from the LED was collimated using a 0.79 NA ACL25416U-A aspheric condenser lens, and images were produced from the infinity corrected objective using an AC254-200-A-ML achromatic doublet (Thorlabs). Images were captured by mono-color CM3-U3-13S2M-CS CCD cameras (Point Grey) controlled over USB 3.0.

### Microscope imaging volume: capillary design

All capillaries used were borosilicate rectangular 5015 VitroTubes (inner cross-section: 0.05 × 1.00 mm, VitroCom). Capillaries were shortened with a diamond scribe and affixed to a black-anodized machined aluminum plate using OD2002 medical epoxy (Epoxy Technology) cured in an oven at 150 °C. Additional OD2002 epoxy was used to secure the capillary ends to the inside of standard silicone tubing and cured at the same temperature, followed by using a layer of 730 medical epoxy (Epoxy Technology) cured at 100 °C to ensure the tube-capillary-plate joint remained rigid to prevent the capillary from snapping. Capillaries were robust to repeated autoclaving. The aluminum plate was affixed to the microscope by mounting on a machined Thorlabs part of the correct dimensions to fit the microscope’s 30 mm cage system.

### Density calibration

To relate the number of cells per image (Fig. 2a, Supplementary Fig. S4) to the density of cells in the continuous-culture device, a culture of *E. coli* expressing dTomato (Cm^R^) constitutively from the chromosome was grown to stationary phase in M63 minimal media with 0.06% glucose and 12.5 μg ml^−1^ chloramphenicol. Samples of this culture, which did not contain aggregates, were diluted to varying concentrations over an approximately 200-fold range in phosphate-buffered saline. A small portion of each sample was plated, with the remainder of the sample put in the continuous-culture device and imaged for 30 minutes. The colonies on the plates were counted 24 hours later, then again after another 24 hours to check for the presence of slow-growing cells. Finally, the number of colonies converted to cell density was plotted against the 30-minute average of the number of cells detected by image processing (Supplementary Fig. S5) and a linear regression was performed on these data which yielded an estimate of the imaging volume: 2.96 ± 0.03 × 10^−7^ ml.

### Microscope imaging line vibration damping

Imaging problems caused by rapid micron-scale liquid oscillations in the microscope imaging line were solved by allowing the lines to sit slightly stretched along aluminum T-slotted extrusion frames mounted to the optical table using special fasteners (McMaster-Carr) and optical posts.

### Image analysis

(Software available at: https://github.com/jmerrtt2/ChemostatImageProcessing/) Using the scikit-image and NumPy packages, all images from the microscope are passed through a nearest-neighbor median filter to remove hot pixels, followed by a bilateral mean filter to smooth the image while preserving any sharp boundaries between regions of different brightness. Images were analyzed using separate methods to detect cells and clumps, and subsequently analyzed to discard low-quality images.

### Feature detection and cell analysis preparation

Images are passed through a difference of Gaussians filter to detect the locations of bright, cell-sized objects. Local subimages of the image are isolated around each detected object. The bottom third of the intensity range of each subimage is discarded as noise, and the remainder of the intensity range is divided into 14 equal-width intensity bins (Supplementary Fig. S6). 14 binary masks are constructed, corresponding to pixels with intensity values that fall within each bin. These masks are subjected to a binary erosion to remove isolated pixels, followed by a binary dilation to smooth edges. Next, all the masks for a subimage are layered, with higher-intensity masks on top, to generate a new 15-color subimage representing a simplified, smoothed approximation of the subregion’s object. Finally, the subimage is divided into regions of connected equal-intensity pixels, and each region is assigned a list of all neighboring regions.

### Subimage analysis and cell identification

The 16-color subimage analysis follows a modified form of Lindeberg’s algorithm^11^. Regions from each subimage which are local maxima, defined as being adjacent to no regions of greater brightness, are designated as blob ‘seeds.’ Any seeds located on the border of the subregion are viewed as representing objects other than those detected by the difference of Gaussians filter, and are treated the same as other seeds for the remainder of the subimage analysis but ultimately discarded. Seeds form blobs by flooding outwards, absorbing neighboring, lower-intensity regions, starting with the brightest regions in the image and ending with those 1 intensity value above background. Regions which would be absorbed by two separate blobs are assigned competitively to whichever initial blob seed is closer to the region’s centroid. After this process finishes, masks are made of each blob other than those with seeds on the image border, and applied against the original image. Finally, a series of features for machine learning are calculated for each blob, including blob size, shape, and intensity. These blob features are subsequently used to train a support vector machine (SVM, scikit-learn: http://scikit-learn.org/) with a hand-curated training set. The output of the SVM determines if each blob is considered as noise, a single cell, or, more rarely, two cells which could not be distinguished as different objects during the subimage analysis, typically because they overlapped.

### Clump detection

Using a free implementation of Tsai’s statistical moment-preserving threshold^12^, a binary mask of the entire image is constructed preserving statistically important objects. Disconnected regions of this mask are collected as possible clumps, and small objects which should be detected as cells are immediately discarded by a size threshold. Next, intensity information corresponding to remaining large images is collected and any objects meeting set intensity thresholds are initially counted as clumps. Finally, suspected clumps are checked against existing cell information, and clumps which substantially overlap with cells are discarded as being locally-dense collections of cells.

### Estimation of clump size

To estimate a lower bound on the number of cells in each clump (aggregate) we computed the average size of planktonic cells (5.2 ± 4.25 μm^2^) and the average size of aggregates (113 ± 98 μm^2^) for a single replicate system from Fig. 2a. Sizes are nominal and were estimated assuming 20x magnification and 3.75 × 3.75 μm pixels for the camera used. Therefore, the aggregates have a cross-sectional area, on average, corresponding to 21.7 cells. This is a lower bound given that the aggregates are three dimensional. To estimate an upper bound we assume aggregates and cells are spheres with radii computed from the average areas listed above. In this case we estimate 52.5 cells per aggregate. In the latter case, when we observe three aggregates per image on average, the aggregates can contain approximately the same biomass as the planktonic population.

### Image and sample quality analysis

Rather than doing further detailed image analysis to detect problems like large bubbles in the imaging region or liquid moving too quickly for cells to be imaged, we rely on statistical tests of detected cell data and abundances to discard low-quality images. First, because cell positions in our images should be drawn from a uniform distribution, we calculate the probability of the detected cell positions in an image being drawn from a uniform distribution and discard images with spatial distributions that are exceedingly unlikely given this assumption (Supplementary Note: Image spatial analysis and quality control). This step removes images which contain large bubbles. Second, denote the number of cells detected in image 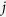 of the 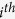 minute of the experiment as 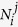, where 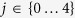. We discard all 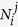 if more than two images in the image set from minute 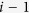 were discarded or if 
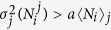
 where 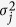 denotes the variance in 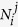 for all *j* for a given minute 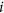, and 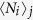 denotes the average across *j*. The factor *a* was determined empirically and was set to 3.2. Although 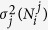 should not be Poisson-distributed due to temporal correlation, acceptable variances due to counting noise still depend on 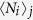, and were determined empirically by plotting 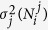 against 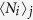 for all *i*. This step removes images where the liquid in the capillary was moving very quickly or accurate imaging was not possible. Finally, we discard data from minute *i* if 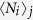 is substantially different from the mean counts over the previous five time points assuming Poisson fluctuations (Supplementary Note: Abundance time variation quality control). This removes time points with unusual problems, including images with large bubbles that were not discarded in previous steps.

### Growth rate analysis

We defined 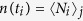. Growth rates are determined by fitting log(*n*(*t*)) using smoothing spline functions from R. Bootstrap aggregation over smoothing spline fits is used to produce a final fit and estimate of fitting uncertainty. Adding the system’s instantaneous dilution rate to the first derivative of this fit yields the instantaneous specific growth rate for the single-cell population (Supplementary Fig. S1). To estimate the number of generations that have occurred up to time *T* in the experiment we compute 
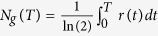
 where *r*(*t*) is the time dependent specific growth rate estimated by spline regression.

### Media and chemicals

All continuous culture experiments used M63 minimal media with 0.04% glucose. Plating was carried out on LB agar plates. For all experiments other than testing the effect of its absence, chloramphenicol was added to both plates and liquid growth media at 12.5 μg ml^−1^.

### Growth conditions

Single colonies were inoculated into fresh M63 media in flasks and grown until visibly dense (~2 days) at 30 °C with shaking at 200 rpm. This culture was diluted 2-fold into fresh media and used to initiate replicate chemostats for an experiment. All experiments were carried out with a stir rate of 800 rpm in systems with automatic temperature control at 30 °C, in a room also environmentally controlled 30 °C (Darwin Chambers), ensuring the culture itself, incoming media, and any bacteria in the imaging line would be held at the same temperature. In batch culture experiments, cultures were simply stirred and pumped through the microscope until the end of the experiment. For the long-term constant-dilution rate (chemostat) experiment, populations were grown at a dilution rate of 0.08 h^−1^ for ~23 days. For all other continuous culture experiments, populations were allowed to acclimate to constant-dilution chemostat conditions over the course of 2 days at a basal dilution rate of either 0.08 hr^−1^, 0.16 hr^−1^, or 0.28 hr^−1^ before the first washout event took place. Washout events then repeated at the same time every day until the end of the experiment.

### Strains

All strains were non-mating Cm-resistant MG1655 derivatives developed by D. Hekstra at Rockefeller University^18^. We worked with three strains in this study. MG1655 Δ*fimA*, Δ*flu*, HK022 att:: (*cat* P_λR_-dTomato) *hsdR*, which expresses the red fluorescent protein constitutively from the chromosome. The Δ*fimA*, Δ*flu* mutations reduce adhesion substantially in this strain. We used a derivative of this strain which did not contain these mutations (MG1655 HK022 att:: (*cat* P_λR_-dTomato) *hsdR*) to test for the possibility that these mutations were responsible for the aggregation we observe. Finally, we used a strain encoding the fluorescent reporter on a plasmid MG1655 Δ*fimA*, Δflu PZS*3R dTomato. This plasmid has a pSC101* origin of replication which results in a copy number of approximately 5. The plasmid strain is substantially brighter, which is necessary for reliable imaging doubling times greater than approximately 3 hours.

## Additional Information

**How to cite this article**: Merritt, J. and Kuehn, S. Quantitative high-throughput population dynamics in continuous-culture by automated microscopy. *Sci. Rep.* **6**, 33173; doi: 10.1038/srep33173 (2016).

## Supplementary Material

Supplementary Information
