# Supplementary material for: Quantitative high-throughput population dynamics in continuous-culture by automated microscopy
Source: Sci Rep. 2016 Sep 12;6:33173. doi: 10.1038/srep33173 (PMC5018735; doi:10.1038/srep33173)
Supplement: Supplementary Information [file srep33173-s1.pdf]

Supplementary information for "Quantitative high-throughput population dynamics in continuous-culture by automated microscopy"

Jason Merritt and Seppe Kuehn

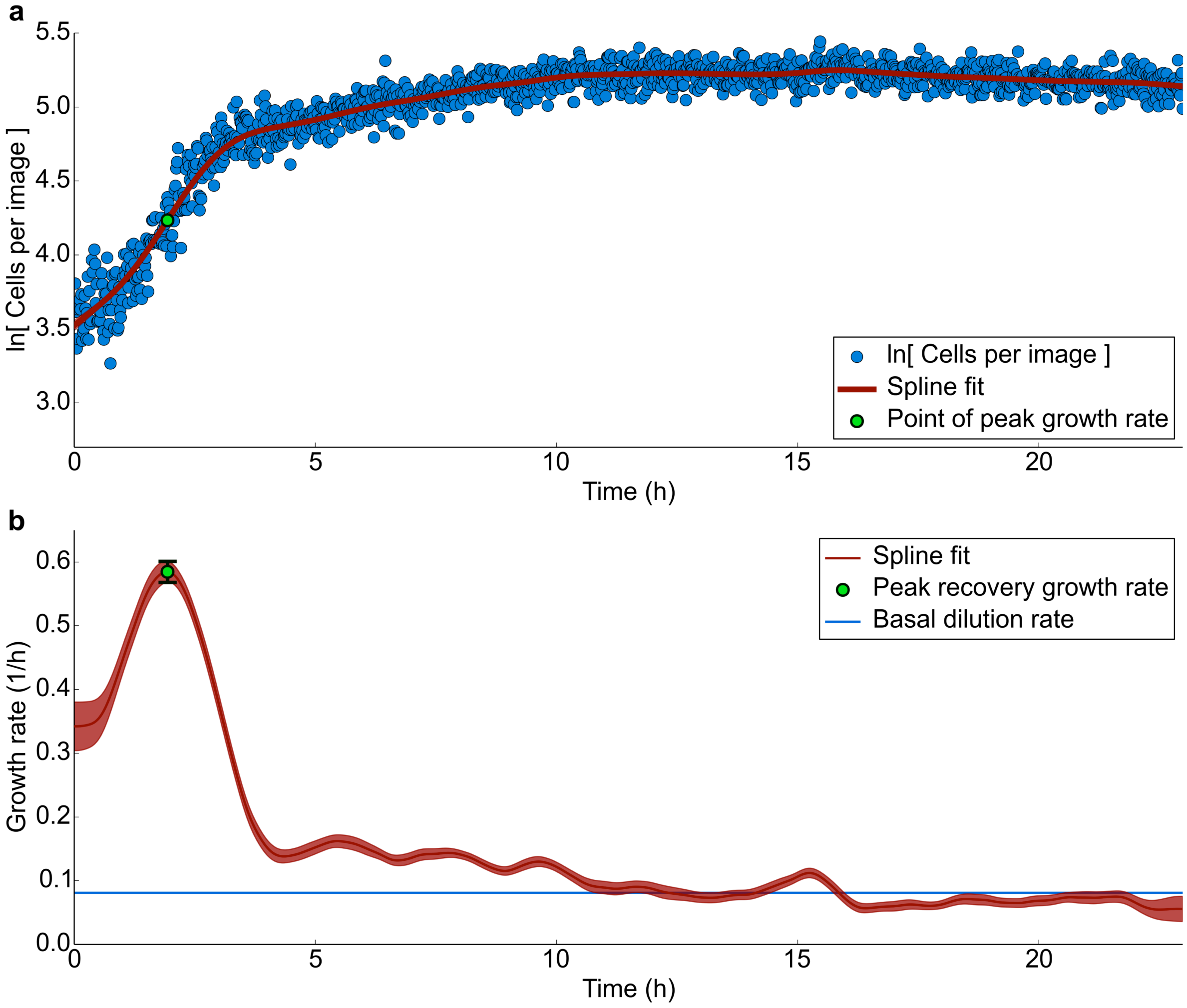

Supplementary Figure S1: Peak recovery growth rate estimation (visual example).

(a) Natural log of number of detected single cells, not including aggregates, per image from a continuous-culture device just after a washout event occurred (washout event ends at time $t = 0$). The red line is a spline fit determined by bootstrap aggregation. **(b)** Note that for exponentially growing populations $\frac{d \ln(N(t))}{dt} = r$ where r is the growth rate. The spline regression results in an estimate of $\frac{d \ln(N(t))}{dt}$ for the fit shown in **a**. The shaded region shows the standard error of the distribution of spline fit derivatives at each time point. The basal dilution rate of the chemostat (the growth rate at steady state) is shown by the blue line.

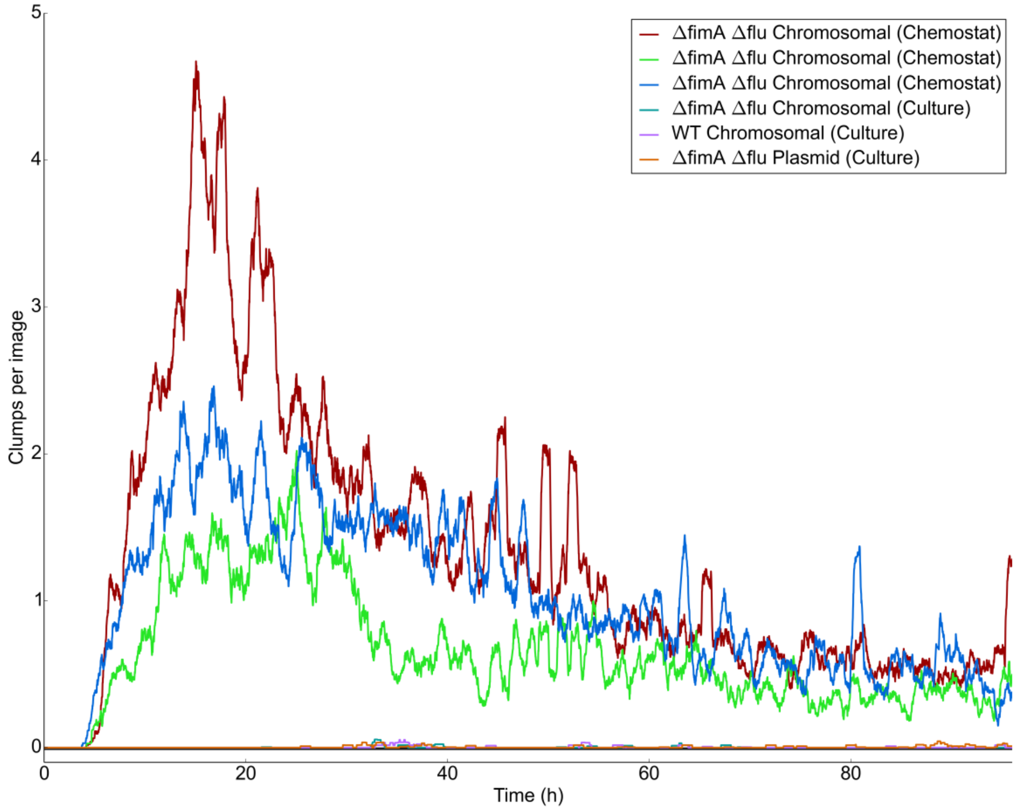

Supplementary Figure S2: Cell aggregation in batch culture vs. chemostat conditions.

Cell aggregation of *E. coli* expressing dTomato constitutively from the chromosome or a plasmid in batch culture and chemostat conditions (D=0.08 h^{-1}, Supplementary Fig. S4). Clumps almost never form in batch culture even after four days, and most of the few clumps detected in batch culture are likely false positives due to overlap and exceptionally bright planktonic cells.

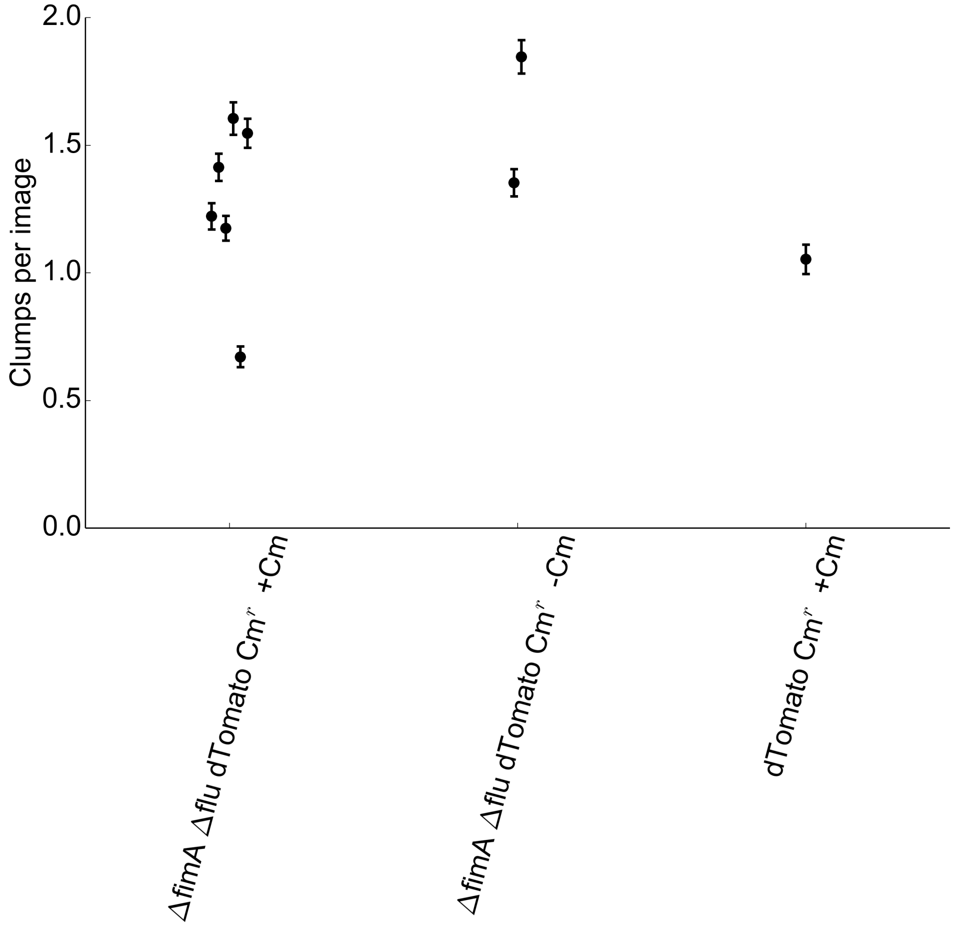

Supplementary Figure S3: Cell aggregation dependence on antibiotic and genotype.

All strains used here are chloramphenicol resistant (Cm^r). "dTomato Cm^r"denotes MG1655, HK022 att:: (*cat* $P_{\lambda R}$-dTomato) *hsdR.* For experiments shown in Fig. 2 of the main text the medium contained chloramphenical to reduce contamination risk. Here we confirm that the presence of this antibiotic did not induce clumping and that the *Δflu* and *ΔfimA* mutations did not cause aggregation. Estimates of average clumps per image determined by averaging across the last 8 hours of a 2 day period of constant dilution at D=0.08 h^{-1}. We do not see a significant change in the amount of cell aggregation depending on the presence of Cm (Welch's t-test, p=0.28) or the *Δflu* and *ΔfimA* mutations (probability of observing number of clumps at least as far from the mean as in dTomato Cm^r +Cm given the observed number of clumps in *Δflu*, *ΔfimA* dTomato Cm^r +Cm is 0.48, two-tailed test). The plasmid strain (MG1655 *ΔfimA, Δflu PZS*3R dTomato)* produces a similar number of aggregates in the same conditions (Fig. 2e), but is not shown here because the increased brightness of the strain artificially raises the number of detected aggregates if the same thresholds are used. The outlier in the *ΔfimA Δflu* +Cm condition was caused by a sudden drop in the number of detected aggregates in one system after ~30 hours (shown in Supplementary Fig. S2)

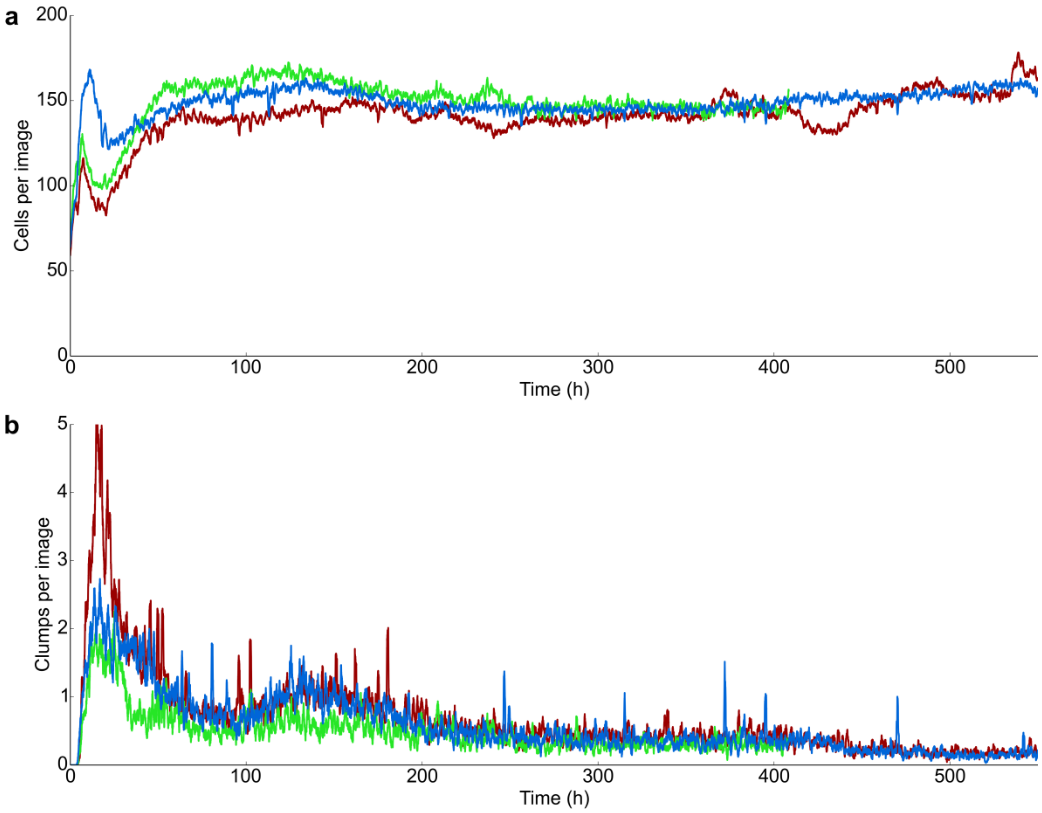

Supplementary Figure S4: Steady-state chemostat experiment.

(a) Single-cell abundances, not including aggregates, from image segmentation in three replicate steady-state chemostats (D = $0.08h^{-1}$) over a 23-day experiment. Data shown is smoothed by a rolling average with a one-hour window. One system was stopped after ~16 days due to imaging problems. **(b)** Clump abundances for three replicate systems during the same experiment shown in **a**. Data shown is smoothed by a rolling average with a one-hour window.

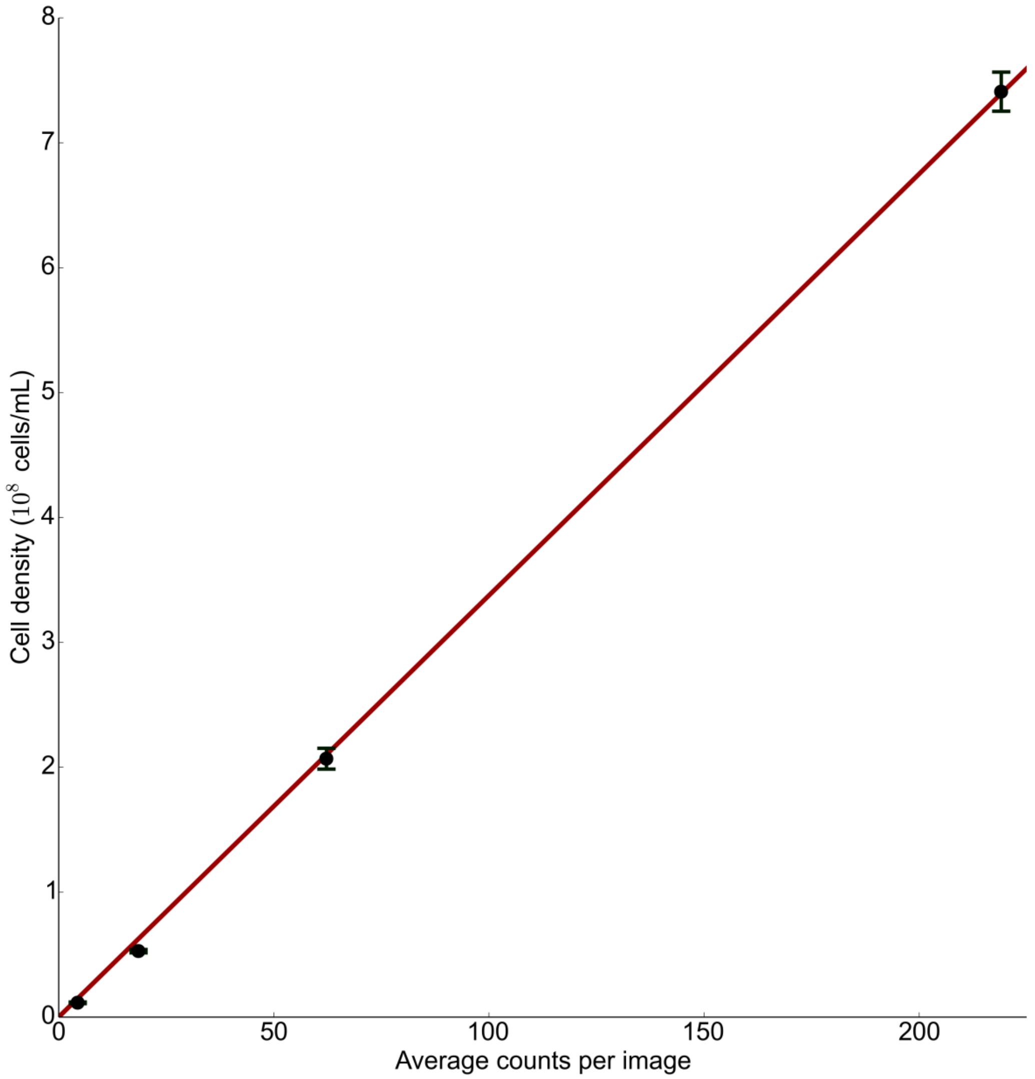

Supplementary Figure S5: Density measurement calibration.

Calibration curve to convert cells/image to a culture density. Counts per image for samples taken from cultures with densities were determined by plating (Methods). By linear regression we calculate a conversion factor of $3.375 \pm 0.027 \times 10^6$ (cells/mL)/(counts/image).

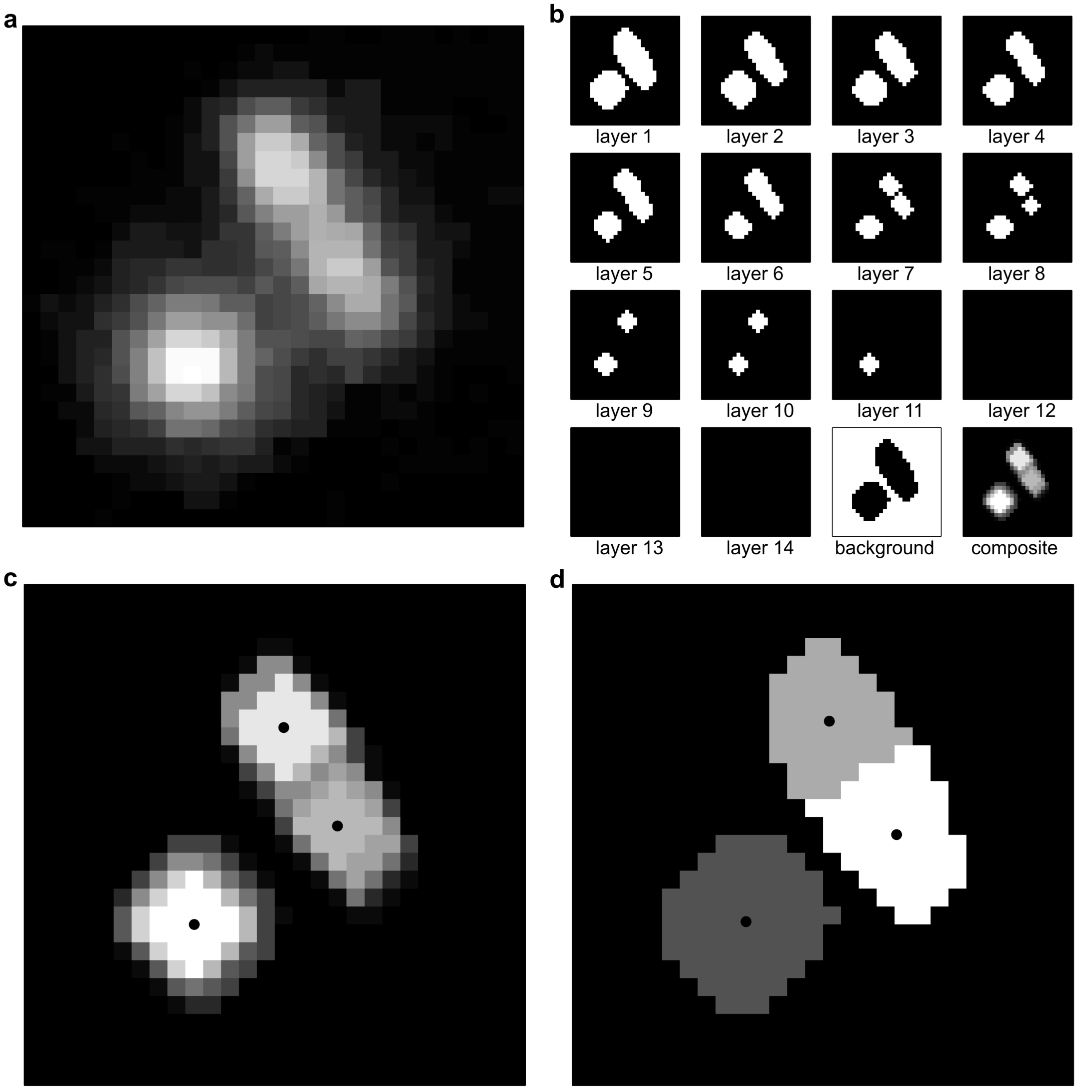

Supplementary Figure S6: Subimage segmentation of detected features in images.

(a) Real subimage data, having undergone a median filter and bilateral mean filter for smoothing. **(b)** Individual and composite masks representing different brightness thresholds. Each individual mask separately undergoes a binary erosion and dilation to remove small features and smooth edges. **(c)** Composite mask, with black dots indicating centers of blob seeds (Methods). **(d)** Blob seeds expanded to create blob masks (black dots indicate mask centroids).

Supplementary Note: Image spatial analysis and quality control.

To evaluate the quality of individual images, we calculate the center of mass of all the cells in frame (μ_x, μ_y) and assume cell locations are uncorrelated and uniformly distributed along both the x and y axes. For a uniform distribution of N cells along the x dimension in an image of discrete width W the expected standard error of the mean is $\sigma_{x,theory}(N) \equiv \sqrt{\frac{W^2-1}{12N}}$. If we assume the center of the image to be the origin and use the Q-function to approximate the odds of having the mean be located at least a distance $|\Delta\mu_x|$ from the center (in either direction) along the x-axis we calculate an approximate probability $P_x(\mu_x, N) = 1 - erf\left[\frac{|\Delta\mu_x|}{\sigma_{x,theory}(N)*\sqrt{2}}\right]$. Calculating the probability along the y-axis the same way, the total probability of getting an image with the center of mass of the cells as far from the center of the image as we actually get is approximated by $P = P_x P_y$. Under this approximation, we discard images with a result extreme enough to have a probability below 0.0001%. This is a lenient threshold; only 1 in 1 million images would be expected to yield a result this extreme based on chance alone. Therefore, images rejected under this threshold alone are nearly always faulty in some way, most often due to containing large bubbles that significantly skew the distribution of cells.

Supplementary Note: Abundance time variation quality control.

Because our image quality control is imperfect, after evaluating the apparent quality of images from individual minutes (Methods) we additionally check the quality of an individual minute's data by comparing it to data from previous images. We find that this processing step removes time points that were not excluded in previous processing steps but where imaging failed. Let $\langle N_i \rangle_j$ denote the average cell counts of all frames j taken during a minute i. Starting with the first five minutes, we keep a rolling set S_i of five average counts from five earlier minutes which passed the criteria specified below, which we define as $S_i = \{\langle N_{k_1} \rangle_j, \langle N_{k_2} \rangle_j, \langle N_{k_3} \rangle_j, \langle N_{k_4} \rangle_j, \langle N_{k_5} \rangle_j\}$ for minutes indexed by $k_1, k_2, k_3, k_4, k_5 < i$.

Each minute's average counts $\langle N_i \rangle_j$ are compared against S_i as described below and combined with the elements of S_i (removing the earliest element of S_i in the process) to create the new set S_{i+1}. If a minute's data fails to pass the criteria stipulated below it is discarded. To prevent rejecting data for long stretches of time a minute's average abundance $\langle N_i \rangle_j$ is allowed into S_{i+1} regardless of whether it passes the criteria defined below if the oldest element of S_i is from at least ten minutes earlier (e.g. $\langle N_{i-10} \rangle_j$).

$\langle N_i \rangle_j$ is compared to the elements of S_i, two standard deviation estimates are calculated: the theoretical Poisson standard deviation $\sigma_{i,theory} = \sqrt{mean(S_i)}$ and the empirical standard deviation $\sigma_{i,experiment} = std(S_i)$. Next, the greater of these two standard deviations $\sigma_i = max(\{\sigma_{i,theory}, \sigma_{i,experiment}\})$ is taken, and $\langle N_i \rangle_j$ is retained if $|\langle N_i \rangle_j - mean(S_i)| < 3\sigma_i$ or, if previous image quality control steps determined the minute's data appeared sufficiently trustworthy, the slightly more lenient condition $|\langle N_i \rangle_j - mean(S_i)| < 4\sigma_i$. In a typical experiment, <5% of the minutes in an experiment are discarded during this step.

Because this algorithm still allows some poor quality data through, particularly when many recent minutes' worth of data had already been discarded, the final data sets are checked by hand. Rapid and apparently impossible density fluctuations (such as a very high cell density dropping to zero in two minutes) are checked by direct comparison to images from the original experiment, and minutes containing images with clear problems such as large but undetected bubbles are discarded to create the final time series. In a typical experiment, fewer than ten such questionable fluctuations need to be checked by hand.
